# The utility of serum biomarkers to detect myocardial alterations induced by Imatinib in rats

**DOI:** 10.1002/prp2.15

**Published:** 2014-03-03

**Authors:** Eugene Herman, Alan Knapton, Jun Zhang, Joel Estis, John Todd, Steven Lipshultz

**Affiliations:** 1 Division of Drug Safety Research, Food and Drug AdministrationSilver Spring, Maryland; 2 Singulex, Inc.Alameda, California; 3 Department of Pediatrics, Mailman Center for Child Development, Leonard A. Miller School of Medicine, University of MiamiMiami, Florida

**Keywords:** Cardiac biomarkers, Imatinib cardiotoxicity, rats

## Abstract

**Background:**

Imatinib (Imb) is a tyrosine kinase inhibitor with cardiotoxic activity (decreases in left ventricular function and congestive heart failure) in patients. Currently, clinical diagnosis of Imb cardiotoxicity relies primarily on evaluation of left ventricular function, Imb also induces cardiac lesions in rats.

**Aims:**

This study, in rats, sought to determine whether monitoring biochemical markers would be a sensitive means to detect Imb-induced changes in cardiomyocyte morphology.

**Materials and methods:**

Groups of male Sprague–Dawley rats were dosed orally with 50, 100, 200 mg kg^−1^ Imb or water daily for 28 days. Tissues and blood samples were collected 24 h after the last dosing. Cardiac biomarkers such as cardiac troponin I (cTnI), cardiac troponin T (cTnT), and fatty acid binding protein 3 (FABP3) were monitored by the Erenna, Elecsys, and Meso Scale immunoassay systems.

**Results:**

Imb caused microscopic myocardial lesions (myofibrillar loss, cytoplasmic vacuolization, and necrosis) at all doses as determined by unbiased histopathology analysis. The severity of the alterations was dose-related with mean lesion scores (based on a scale of 0–3) of 1.2 (50 mg kg^−1^), 2.1 (100 mg kg^−1^) and 2.9 (200 mg kg^−1^). However, the increases in cTnI, cTnT, and FABP3 levels were noted primarily in high-dose Imb treated animals.

**Discussion and conclusion:**

The occurrence of myocardial alterations in animals without consistent changes in cardiac troponin and FABP3 concentrations raises questions regarding the utility of these biomarkers as early indicators of Imb-induced cardiotoxicity. Due to limited numbers of animals the reasons for this discrepancy could not be determined.

## Introduction

Tyrosine kinase inhibitors (TKI) are a relatively new class of therapeutic agents that are intended to act on specific pathways that are overexpressed in certain types of neoplastic cells (Sawyers 2003). Most currently utilized TKI-type drugs are targeted to interfere with kinase pathways that, when overexpressed, facilitate abnormal tumor cell growth (Krause and Van Etten 2005). Development of TKI agents, such as Imatinib (Imb), has been useful in the treatment of neoplasms whose pathogenesis was associated with abnormalities in tyrosine kinase regulation. However, these same pathways have also been implicated in maintaining the viability of cells in normal tissues such as the heart. Interference with these tyrosine kinases is thought to be responsible for adverse myocardial effects (deterioration in cardiac function and/or congestive heart failure) that have been reported during clinical use of Imb and other TKI agents (Kerkela et al. 2006; Force et al. 2007). Hartmann et al. (2009) found a low overall incidence of TKI-induced cardiotoxicity except in elderly patients with preexisting conditions. The actual clinical incidence of this toxicity may be underestimated because in many studies the procedures utilized to evaluate cardiac function had limited diagnostic sensitivity (Force and Kolaja 2011).

A common clinical paradigm for assessing the status of cardiovascular function during chemotherapy consists of monitoring changes in left ventricular function by techniques such as echocardiography or multigated acquisition scanning (Raschi and DePonti 2012; Todaro et al. 2013). In many instances, these procedures detect normal cardiac function even though a certain degree of cardiac cell injury or loss has occurred (Ganz et al. 1996; Eidem 2008; Altena et al. 2009; Monsuez 2012). As a result patients receiving further chemotherapy may be subjected to the risk of developing additional cardiac injury and/or heart failure. There has been increasing interest in exploring additional means to detect chemotherapy-induced myocardial injury such as monitoring serum biomarkers that are indicative of cardiac injury (e.g., cardiac troponin I (cTnI) and cardiac troponin T (cTnT), myoglobin, fatty acid binding protein 3 (FABP3), and creatine kinase) and/or heart failure (B-type natriuretic peptide [BNP]; Gaze and Collinson 2005; Force and Kolaja 2011). Previously, these biomarkers have provided information regarding myocardial damage elicited by both ischemic and nonischemic causes (Gaze and Collinson 2005). At present, information regarding the potential utility of these cardiac biomarkers for the early diagnosis and continuing prognostic assessment of TKI-induced cardiotoxicity is limited (Ederby et al. 2012). This study utilized a rat model to determine whether monitoring serum concentrations of cardiac troponins and/or FABP3 would allow detection and differentiation in the degree of cardiac injury induced by increasing doses of Imb. In particular, the study included monitoring of serum cardiac biomarker concentrations by means of several immunoassay systems in conjunction with a corresponding detailed evaluation of myocyte morphology.

## Materials and Methods

All procedures involving animals were approved by the Institutional Animal Care and Use Committee, Center for Drug Evaluation and Research, Food and Drug Administration, and complied with the guidelines for the Institute of Laboratory Animals Resources Guide for the Care and Use of Laboratory Animals (National Research Council, eighth edition, 2011).

Adult male Sprague–Dawley (SD) rats, 10–12 weeks of age, were obtained from Harlan Laboratories (Indianapolis, IN). Animals were housed individually and given rodent chow (Lab Diet 5002) and water ad libitum. Experiments began after a week of acclimation.

The study involved the daily administration of Imb at doses of 50, 100, or 200 mg kg^−1^ for 28 days. The 50 and 100 mg kg day^−1^ doses had been utilized in a previous study and found to cause myocardial lesions of minimal to mild severity (Herman et al. 2011). The lowest dose of Imb selected (50 mg kg^−1^ day^−1^) (295 mg m^−2^ day^−1^) was in the range of dose used in clinical treatment regimens (400–800 mg day^−1^ or 228–456 mg m^−2^ day^−1^ based on a weight of 70 kg).

### Study procedures

Imb methanesulfonate salt was obtained from LC Laboratories (Woburn, MA). Twenty SD rats were divided into four experimental groups of five animals each. Groups 1–3 received 50, 100, and 200 mg kg^−1^ Imb, respectively, and the fourth or control group received distilled water at a volume equivalent to the 200 mg kg^−1^ per dose (0.4 mL 100 g body weight^−1^).

Imb was dissolved in distilled water (54.05 mg mL^−1^) and administered by gavage in dose volumes of 0.1, 0.2, and 0.4 mL 100 g body weight^−1^ daily for 28 days. Each animal was observed daily for overt signs of toxicity and weighed twice a week.

### Study termination procedures

Animals were anesthetized with isoflurane 24 h after the final dose of Imb was given. The inferior vena cava was exposed through a midline abdominal incision. Terminal whole blood samples for hematological, clinical chemistry, and biomarker analysis were collected through a 19-G Venocath inserted into the vena cava. Blood samples collected for plasma and serum were immediately centrifuged at 3000 *g* for 10 min and frozen at −80°C until analyzed. Anesthetized animals were euthanatized by exsanguination and induction of a pneumothorax.

### Pathologic evaluation

The heart, kidneys, and portions of the liver and small intestine were excised and fixed in 10% neutral buffered formalin. Hearts were embedded in glycol methacrylate plastic resin. Sections (1-μm thick) of the plastic-embedded left ventricular heart tissue were stained with alkaline toluidine blue. The noncardiac tissues and some cardiac tissues were embedded in paraffin and stained with hematoxylin-eosin.

All toluidine blue-stained plastic sections of cardiac tissues (2–4 sections/heart) were examined by a research pathologist. The evaluation was blinded to treatment and any related biomarker data. The severity of myocardial lesions was scored on a semiquantitative scale of 0–3 under light microscopy (Billingham 1991), but necrosis of myocytes was also added to the scale. Thus, the scoring system used to access cardiac lesion severity in this study was based on the proportion of muscle cells showing myofibrillar loss, cytoplasmic vacuolization and necrosis: 0, none or a negligible percentage; 1, less than 5%; 1.5, between 6% and 15%; 2, between 16% and 24%; 2.5, between 26% and 35%; and 3, greater than 35%.

### Sample analysis

#### Clinical chemistry and hematology

Terminal serum clinical chemistry analysis was performed on the VetScan model #200-1000 using the Comprehensive Diagnostic and Large Animal rotors (Abaxis, Inc., Union City, CA). Blood cell counts and other hematological values were determined using the VetScan HMT (Abaxis, Inc.).

### Serum biomarker analysis

#### cTnT *assay*

Blood samples were centrifuged (3000 *g* for 15 min) and the serum was frozen at −40ºC until assayed for concentrations of cTnT (Elecsys Stat; Roche Diagnostics, Indianapolis, IN) the laboratory of Children's Hospital, Harvard Medical School, Boston, MA. Technicians were blinded to the treatment. This assay was used in a previous study (Herman et al. 2006).

#### cTnI Assay

Blood samples were processed as described above. Terminal serum concentrations of cTnI were measured with the ultrasensitive Erenna Immunoassay System (Todd et al. 2007) (Singulex, Alameda, CA). The assay was used in a previous study from this laboratory (Herman et al. 2011).

#### Multiple cardiac biomarker assay

Following manufacturer's instructions, the cardiac biomarkers cTnI, cTnT, and FABP3 present in the serum were measured using proprietary rat kits on the Meso Scale Discovery (MSD) electrochemiluminescence platform (Meso Scale Discovery, Gaithersburg, MD). Use of this assay in rats has been reported by Tonomura et al. (2012).

### Statistical analysis

The Kruskal–Wallis test for non-normally distributed data was used to assess differences in the myocardial lesion scores between the various treatment and control groups. The Tukey–Kramer multiple comparisons test was used to assess group-related differences in body weight, hematologic values, clinical chemistry values, and cardiac biomarker concentrations. All data met the assumptions of the tests used to analyze them. Alpha was set at 0.05 and all tests were two-tailed. The InStat (GraphPad Software, Inc., San Diego, CA) statistical software package was used for all analyses.

## Results

### General toxicity and body weight gain

Two rats from the high-dose group died within 2 h following gavage during the fourth week of dosing. These deaths were attributed to an oral dosing error. Blood samples were not available but tissues from these animals were included in the analysis.

The final body weights of control animals and animals in the 50 and 100 mg kg^−1^ treatment groups were not significantly different from each other (Table 1). These animals had gained an average of 8 and 20% in body weight, respectively. Vehicle control animals gained an average of 18% weight over the 4-week course of the study. The mean weight of the group that received the 200 mg kg^−1^ dose was lower than that in the other groups and these animals lost an average of 5% in body weight over the 4-week duration of the study (Table 1).

**Table 1:** Body weight, hematologic, and clinical chemistry values (mean ± SD) in SD rats treated daily with Imatinib for 28 days

Imatinib dose (mg kg^−1^)	Final Wt (g)	%Change in Wt (g)	ALT (μ L^−1^)	Albumin (g dL^−1^)	Glucose (mg dL^−1)^	WBC (×10^3^)	RBC (×10^3^)	Hct (%)	Hb (g dL^−1^)
50	405 ± 28	8	55.6 ± 7.5	4.5 ± 0.3	186 ± 12	8.3 ± 1.6	7.0 ± 0.6^1^	44.5 ± 3.2	12.6 ± 1.2
100	457 ± 22	20	69.0 ± 16.2	4.7 ± 0.2	178 ± 15	8.3 ± 1.1	6.7 ± 0.3^1^	42.0 ± 1.5^1^	12.1 ± 0.2
200	360 ± 26	−5^1^	3943 ± 207^1^	8.7 ± 1.6^1^	119 ± 82	8.9 ± 1.7	6.3 ± 2.4^1^	37.3 ± 8.3	10.9 ± 3.4
Control	444 ± 11	18	45.4 ± 2.7	4.1 ± 0.1	199 ± 22	10.0 ± 2.1	8.4 ± 0.3	46.4 ± 2.9	13.7 ± 0.6

ALT, alanine aminotransferase.

1 Significantly different from control group, *P* < 0.05, Tukey–Kramer multiple comparisons test.

### Hematology and clinical chemistry measurements

All doses of Imb caused slight but statistically significant decreases in the red blood cell count (Table 1). The hematocrit (%) also declined but the decrease was significant only at the 100 mg kg^−1^ dose (Table 1). The serum concentrations of alanine aminotransferase (ALT) (μ L^−1^) and albumin (g dL^−1^) were significantly increased in animals treated with the 200 mg kg^−1^ dose of Imb (Table 1).

### Gross anatomic changes

At necropsy (1 day after the end of the 4-week dosing period), no visible macroscopic alterations were observed in tissues such as the heart, liver, kidney, spleen, and small intestine from Imb-treated or control rats.

### Myocardial histopathology

**Figure 1:**
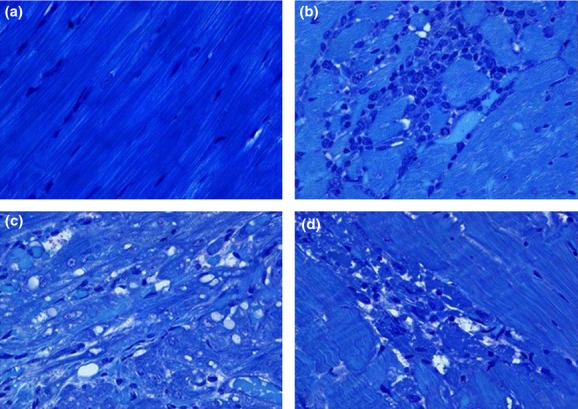
(A–D) Light micrographs showing cardiac alterations in left ventricle from male Sprague–Dawley rats treated orally with water (A, B) or Imatinib (Imb) at doses of 200 mg kg^−1^ (C, D) for 28 days. (A) The cardiac myofibrils from water-treated rats showed regular arrangement of thick and thin myofilaments. (B) Spontaneous background cardiac myocyte necrosis with mixed mononuclear cell infiltration in a water-treated control rat. (C) Focal myofibrillar loss and formation of varied-sized cytoplasmic vacuoles in the myocardium of a rat treated with 200 mg kg^−1^ Imb. (D) Focus of intact and fragmented necrotic cardiomyocytes in association with cytoplasmic vacuoles in the myocardium from a rat treated with 200 mg kg^−1^ Imb. Glycol methacrylate-embedded, alkaline toluidine blue-stained, 1-μm thick plastic sections. Original magnification 630×.

Light microscopic examination of hearts from the five water-treated control rats showed cardiac myocytes with regular arrangement of thick and thin myofilaments (Fig. 1A). However, evidence of a spontaneously occurring myocardial injury was noted in three of these five hearts. The characteristic alteration noted in the affected areas was myocyte necrosis with associated inflammatory cell infiltration (Fig. 1B). Reagan et al. (2013) have also detected similar spontaneous background lesions in rats. Inflammatory cells were generally not present in the necrotic areas observed in the hearts from Imb-treated rats. As a result, the spontaneous necrotic lesion was considered to be nondrug-related and was not included in the scoring system used to evaluate the overall severity of Imb-induced myocardial alterations.

All animals exposed to Imb developed myocardial lesions. The most frequent alterations were cytoplasmic vacuolization and myofibrillar loss (Fig. 1C). Myocardial necrosis was also observed in cardiac tissues from Imb-treated rats. In some instances, cytoplasmic vacuoles were observed within the affected necrotic myocytes. In contrast to the spontaneously occurring myocyte necrosis described above in the control animals, these areas of Imb-induced necrosis contained few, if any associated inflammatory cells (Fig. 1D). The extent of myofibrillar loss, cytoplasmic vacuolization, and necrosis increased with the dose of Imb (Table 2). Of five hearts from animals given 50 mg kg^−1^ Imb, three had minimal lesion scores of 1.0 and two had mild scores of 1.5. With higher doses myocardial lesions were more extensive (lesion scores ranged from 2.0 [moderate] to 3.0 [severe]) in the hearts of rats treated with 100 or 200 mg kg^−1^ Imb (Table 2). The myocyte lesions induced by the 200 mg kg^−1^ Imb dose were significantly more severe than those occurring at either the 100 or 50 mg kg^−1^ doses (*P* < 0.05). Likewise, the myocardial alterations induced by the 100 mg kg^−1^ dose were significantly more severe than those caused by the 50 mg kg^−1^ dose of Imb (*P* < 0.05) (Table 2).

**Table 2:** Severity of cardiac lesions in male Sprague–Dawley rats treated daily with Imatinib for 28 days

		Drug-induced lesion severity score					
		
Imatinib (mg kg^−1^)	*n*	0	1.0	1.5	2.0	2.5	3.0
50	5^1^	0	3	2	0	0	0
100	5^1^,^2^	0	0	0	3	2	0
200	5^1^,^2^,^3^	0	0	0	0	1	4
Control	5	5^4^	0	0	0	0	0

1 Lesion scores significantly greater than those of the control group (*P* < 0.05).

2 Lesion scores significantly greater than those of SD given 50 mg kg^−1^ Imb (*P* < 0.05).

3 Lesion scores significantly greater than those of SD given 100 mg kg^−1^ Imb (*P* < 0.05) by Kruskal–Wallis test for non-normally distributed data.

4 Three rats had spontaneous lesions not typical of Imatinib-induced cardiac alterations.

### Myocardial biomarkers

#### Serum cTnI

cTnI, measured by the Erenna ultrasensitive immunoassay, was detected in all control animals with a mean concentration of 8 ± 6 pg mL^−1^ (Table 3). The two highest values in the control group (19.9 and 13.0 pg mL^−1^) were from two of the three animals found to have spontaneous necrotic lesions. cTnI was also detected in animals treated with Imb. Animals dosed with 50 mg kg^−1^ Imb had myocardial lesion severity scores of 1.0–1.5 with a mean cTnI concentration (5 ± 2 pg mL^−1^), which was within the range of the control animals. Lesions were more severe in animals given the 100 mg kg^−1^ dose of Imb (lesion scores of 2.0–2.5). An elevated concentration of cTnI (353 pg mL^−1^) was detected in only one of the five animals in this treatment group (lesion score of 2.5). High values in cTnI (MSD) and cTnT (Elecsys) were also noted for this animal. As a result, this value in all three assays was considered an outlier and not used in calculating the mean concentration at the 100 mg kg^−1^ Imb dose level. The mean cTnI concentration as detected by the Erenna assay for the other four animals was 7.8 ± 2.2 pg mL^−1^. cTnI was not detectable by the MesoScale assay system in four of five of the 100 mg kg^−1^ or the entire 50 mg kg^−1^ treatment groups or the control animals (Table 3). The concentration of cTnI was elevated to 139 pg mL^−1^ in one animal dosed with 100 mg kg^−1^ Imb (also mentioned above). All animals treated with the 200 mg kg^−1^ dose of Imb had increased serum levels of cTnI as measured by either the Erenna system (mean = 333 ± 198 pg mL^−1^) or the MSD system (323 ± 265 pg mL^−1^) (Table 3).This group of animals also had the most severe myocardial lesions (lesion scores of 2.5–3.0).

**Table 3:** Mean cardiac lesion severity and mean serum cardiac troponin I, cardiac troponin T, and fatty acid binding protein 3 concentrations (±SD) in male Sprague–Dawley rats treated daily with Imatinib for 28 days

Imatinib dose (mg kg^−1^)	*N*	Mean Imatinib lesion score	cTnI (pg mL^−1^) Erenna	cTnI (pg mL^−1^) MSD	cTnT (pg mL^−1^) Elecsys	cTnT (pg mL^−1^) MSD	FABP3 (ng mL^−1^) MSD
50	5	1.2^1^	5.0 ± 2.0	0	0	0	3.0 ± 1.0
100	5	2.2^1^	7.8 ± 2.2^2^	0^2^	0^2^	0	4.0 ± 1.0
200	3	2.9^1^	333 ± 198^4^	323 ± 265	716 ± 889	116 ± 269^3^	184 ± 283
Control	5	0^1^	8.0 ± 6.0	0	0	0	4.9 ± 1.4

1 Mean of five animals.

2 Mean of four animals (a single value of 353 pg mL^−1^ cTnI, 139 pg mL^−1^ cTnI and 151 pg mL^−1^ cTnT from one animal was excluded from the calculation of mean concentrations for the Erenna, MSD, and Elecsys assays).

3 Mean of three animals (a single value of 466 pg mL^−1^ was included in the calculation).

4 Significantly different from control group and groups treated with 50 and 100 mg kg^−1^ Imatinib, *P* < 0.05, Tukey–Kramer multiple comparisons test.

#### Serum cTnT

The concentration of cTnT measured with the Elecsys immunoassay system was below the level of detection in all control animals and those given 50 mg kg^−1^ Imb. Only one animal in the group dosed with 100 mg kg^−1^ Imb (mentioned previously) had a measurable cTnT concentration (151 pg mL^−1^). The Elecsys assay detected elevated levels of cTnT in all animals treated with the 200 mg kg^−1^ dose of Imb (716 ± 889 pg mL^−1^) (Table 3). The MSD assay detected an elevated serum cTnT concentration in only one animal that was dosed with 200 mg kg^−1^ Imb (466 pg mL^−1^). The high-dose group average including this one value was 116 ± 269 pg mL.

#### Fatty acid binding protein 3

FABP3, as monitored by the MSD assay was detected in all animals. No significant changes in serum levels of FAPB3 were observed in the groups of animals that received 50 or 100 mg kg^−1^ Imb (Table 3). One animal that was treated with 200 mg kg^−1^ Imb was found to have a high concentration of FABP 3 (511 ng mL^−1^). This same animal also had elevated levels of both cTnI and cTnT as detected by other assay systems. The remaining two animals in the 200 mg kg^−1^ Imb treatment group had much lower concentrations of FABP3 (20–22 ng mL^−1^) (Table 3). It should be noted that these values of FABP 3 were higher than those detected in animals from the 100 and 50 mg kg^−1^ treatment groups (4.0 ± 1.0 and 3.0 ± 1.0 ng mL^−1^, respectively) and the control group (4.9 ± 1.4 ng mL^−1^).

### Pathologic evaluation of non cardiac tissue

No treatment-related alterations were observed in the small intestine. However, mild tubular dilatation and luminal proteins were observed at all doses of Imb in the kidney. Occasional small foci of hepatic inflammation were observed in 1/5 and 3/6 animals given 50 or 100 mg kg^−1^ Imb, respectively. We were not able to evaluate the liver tissues from animals that received the 200 mg kg^−1^ dose of Imb.

## Discussion

Recent studies have found that cardiac biomarkers such as troponins can be used to monitor cardiac alterations occurring in patients undergoing anthracycline chemotherapy. The increases in serum concentrations of cardiac troponins detected have provided evidence of early doxorubicin-initiated myocardial alterations (Horacek et al. 2008). Cardinale et al. (2004) reported an early increase in cTnI preceded left ventricular dysfunction in doxorubicin-treated patients. Information as to whether monitoring cardiac biomarkers might be useful in detecting TKI-induced myocardial alterations is limited. Cardinale et al. (2010) found that an increase in cTnI concentration was the best predictor of traztuzumab-induced cardiotoxicity. In a phase 1 study, Ederby et al. (2012) noted that among treatment with various targeted agents, elevations in cTnI occurred most frequently in those patients given anti-angiogenic compounds. Schmidinger et al. (2008) detected elevations in cTnI in approximately 36% of patients who experienced a cardiovascular event while being treated with sunitinib or sorafenib. In contrast, Chu et al. (2007) found that increases in cTnI concentrations were not necessarily predictive of which patients given sunitinib would ultimately develop congestive heart failure or a decline in left ventricular ejection fraction (LVEF).

In this study, we sought to explore the utility of monitoring recognized serum biomarkers such as cardiac troponins and FABP3 as a means of identifying Imb-induced myocardial toxicity in male SD rats. This relationship could be evaluated in detail because the doses of Imb utilized caused myocardial alterations (myofibrillar loss, cytoplasmic vacuolization, and necrosis) in all treated animals (Table 2). As a result, it was possible to derive a semiquantitative lesion severity score for each treated animal. The overall cardiac lesion scores tended to increase in severity in association with increasing Imb doses. Minimal-to-mild alterations (lesion severity scores of 1.0 and 1.5) were detected in the hearts from animals given the lowest dose (50 mg kg^−1^). Lesions of moderate to marked (lesion scores of 2.0 and 2.5) severity were found in the hearts from animals dosed with the 100 mg kg^−1^ dose while the highest dose of Imb (200 mg kg^−1^) induced mainly severe myocardial alterations (lesion severity scores of 3.0).

The characteristics of the myocardial alterations induced by Imb in this study were similar to those found in an earlier study that also included hearts from Imb-treated rats (Herman et al. 2011). In the previous study, we monitored only cTnI concentrations with an ultrasensitive assay. A very modest dose-related increase in cTnI was found in Imb-treated SD rats (50 or 100 mg kg^−1^). However, because of the large variability in individual cTnI values, the differences in mean concentrations between all groups (treated and control) were not significantly different from each other. In this study we sought to further determine whether biomarker levels were reflective of Imb-induced myocardial injury. To insure as complete an evaluation as possible, a variety of immunoassays were used to monitor serum concentrations of the selected cardiac biomarkers (cTnI, cTnT and FABP3) in each animal.

The biomarker data obtained from troponin immunoassays indicated that significant increases in concentrations of cardiac troponins do occur following treatment with Imb (Table 2). However, in spite of the presence of lesions at all doses, significant increases in cardiac troponin concentrations occurred consistently (except in one animal dosed with 100 mg kg^−1^) only at the highest Imb dose (200 mg kg^−1^). It should be noted that the assays were not of equal sensitivity as evidenced by the fact that the MSD assay detected the presence of cTnT in only one of the treated animals (200 mg kg^−1^ Imb) whereas the Erenna immunoassay detected cTnI in all animals (control and treated animals). The observed increase in cardiac troponin concentration after treatment with only the high dose of Imb was a consistent finding with the Elecsys cTnT and the MSD cTnI immunoassays as well as the ultrasensitive Erenna cTnI immunoassay.

White (2011) has summarized at least six different pathogenic mechanisms thought capable of facilitating the release of troponins from cardiac cells. Among such mechanisms, both necrosis and apoptosis are cited as important cellular death processes that can increase the serum concentration of cardiac troponins (White 2011). Previously, these types of myocyte alterations were identified in hearts from Imb-treated rats (Herman et al. 2011). Similar changes in myocyte morphology were likewise, observed in this study. Nonlethal changes in cell viability can also influence cardiac troponin release by altering cell membrane permeability (White 2011). Normal myocyte cell turnover is a continuing activity that promotes the release of small amounts of cardiac troponins into the circulation (White 2011). It is possible that some of these processes were more active at the higher dose of Imb.

The Imb-induced myocyte alterations noted in this study appear to be similar, in certain aspects, to those occurring in the myocardium of animals treated chronically with doxorubicin. The doxorubicin-induced myocyte alterations consist primarily of intracellular vacuolization and loss of myofibrils (Herman et al. 1985). In this study somewhat analogous myocyte alterations were noted in rat hearts after treatment with Imb. However, in spite of the apparent similarities in lesion morphology, the pattern of changes in cardiac troponin concentrations were not entirely comparable. For example, doxorubicin-induced myocardial alterations were associated with persistent dose-related elevations in serum concentrations of cTnT (Herman et al. 1999). In contrast, the presence of even moderately severe Imb-induced cardiac lesions, as noted with the 100 mg kg^−1^ dose, was not associated with any significant change in the serum concentrations of either cTnI or cTnT.

The time course of troponin elevations is an important characteristic of myocardial injury. Acute cardiotoxic activity, such as induced by isoproterenol, occurs with a rapid large increase in serum cardiac troponin concentration that reaches a peak within a few hours and then subsides to baseline by 24–48 h post exposure (Herman et al. 2006). In contrast, doxorubicin causes small increases in cTnT concentrations that are persistent and can be detected for up to 1 week after the end of 12 weeks of weekly dosing (Herman et al. 1999). This type of biomarker release pattern is thought due to an ongoing release of bound cTnT from the contractile apparatus presumably as a result of the continuing adverse effects of doxorubicin on myocyte viability. In this study, high serum concentrations of troponin were detected 1 day after the end of 4 weeks treatment with 200 mg kg^−1^ of Imb. Within that group, the increased troponin levels occurred in conjunction with observable myocardial lesions. An important disconnect was noted at the 50 and 100 mg kg^−1^ Imb doses. At these doses cardiac lesions were also noted but without accompanying elevations in troponin concentrations. It is possible that at the lower Imb doses, limitations of quantity and timing of release restrict the presence of measurable troponin at the single time point selected in this study.

This study expanded the evaluation of potential useful Imb cardiotoxicity biomarkers to include FABP3. Myocytes contain high concentrations of this small unbound cytosolic protein. Because of the cytosolic location, FABP3 is rapidly released into the circulation following cardiac injury and has been regarded as an early sensitive biomarker of acute coronary syndrome (Haltern et al. 2010). FABP3 has also been examined as a diagnostic indicator for myocardial ischemia in situations without overt necrosis (Tambara et al. 2004). Experimentally, concentrations of FABP3, as monitored by the MSD immunoassay, have been found to increase in rats given certain types of acute cardiotoxic compounds (Tonomura et al., 2013). Previously, Hasic et al. (2011) and Clements et al. (2010) reported a good correlation between isoproterenol-induced myocardial histological changes and elevations in serum levels of H-FABP. These investigators concluded that H-FABP was a good early marker but is less sensitive and specific than the cardiac troponins for detecting acute myocardial injury in rats. In this study, the pattern of changes in serum concentrations of FABP3 in SD rats, following treatment with Imb, was similar to that observed for the cardiac troponins. The concentrations of FABP3 detected in SD rats after treatment with the 50 or 100 mg kg^−1^ doses of Imb were no different than the control concentrations. Increases in FABP3 levels were noted only in those animals given the highest dose of Imb (200 mg kg^−1^). A comparison of the magnitude of increases in concentrations among the various biomarkers (at the high Imb dose) indicates that the fold change for cTnI and cTnT was considerably greater than that for FABP3. Rapid renal clearance has been cited as a factor that could compromise the diagnostic utility of FABP3 for any but acute cardiac injury (Haltern et al. 2010). As this study included only one time point it was not possible to determine whether renal clearance could have been a limiting factor. Regardless, the findings of this study tend to indicate that FABP3 is of limited usefulness as a sensitive indicator of Imb-induced cardiotoxicity.

In this study, Imb was found to exert certain non cardiac toxic effects including a decrease in RBC, and at the highest dose, increases in serum concentrations of ALT and albumin. Myelosuppression has been reported previously and is considered a direct pharmacological action exerted by Imb (Cohen et al. 2002). Elevation of liver enzymes and hepatic alterations have also been observed as a toxic effect in both preclinical and clinical studies (Cohen et al. 2002). We were not able to evaluate the tissue from the animals receiving the high dose of Imb and were therefore, not able to relate the elevation in ALT to hepatic injury.

## Conclusions

In summary, the current experiments sought to determine whether certain known myocyte proteins could be used as reliable biomarkers of Imb-induced cardiotoxicity. Rats treated with Imb developed dose-dependent myocardial alterations (identified by light microscopic histopathological evaluation) and increased levels of cardiac biomarkers. Measurement of biomarker concentrations (cTnI, cTnT, and FABP3) was performed by several different immunoassay systems and except for one animal treated with 100 mg kg^−1^ Imb, all showed that the biomarkers were elevated only in animals that received the highest dose of Imb (200 mg kg^−1^). This study identified the lack of a meaningful dose-related association between the levels of cardiac troponins and FABP3 and myocardial lesions, a finding which may preclude the use of these biomarkers as early indicators of Imb-induced cardiotoxicity. Additional studies, with larger treatment groups, will be needed to clarify this question.

### Limitations

In this study, biomarkers were evaluated at only one time point (24 h after final dosing), Even though the morphology of the lesions at this time point indicated chronic injury a more comprehensive timeframe for biomarker analysis would be informative to make sure that some important information was not missed. All animals treated with 100 mg kg^−1^ Imb developed cardiac lesions. However, increases in cardiac biomarkers were detected in only one animal of these animals. A comprehensive evaluation of the hearts from this treatment group might have revealed the reason for the differential biomarker release from only one animal. The number of rats utilized in the study was limited. An increase in the number of animals evaluated in both control and treatment groups would provide a more robust determination concerning the overall predictive value of these cardiac biomarkers with this type of cardiac injury. Cardiac troponin and FABP3 are examples of myocyte injury/death biomarkers. There are also myocyte stress biomarkers, extracellular matrix biomarkers, and neurohormonal biomarkers (Van Kimmenade and Januzzi 2012) that singly or in combination might be more effective at detecting Imb cardiotoxicity.

## Abbreviations

ALT: alanine aminotransferase
BNP: B-type natriuretic peptide
cTnI: cardiac troponin I
cTnT: cardiac troponin T
FABP3: fatty acid binding protein 3
Imb: Imatinib
SD: Sprague–Dawley
TKI: tyrosine kinase inhibitors
